# Love Through a Distorted Lens: The Role of Self-Objectification in Interpreting Ambiguous Female–Male Interactions as Romantic among Women

**DOI:** 10.1007/s10508-025-03358-1

**Published:** 2026-01-13

**Authors:** Yijia Dong, Xijing Wang, Shuning Pan, Lei Cheng

**Affiliations:** 1 Department of Social and Behavioural Sciences, City University of Hong Kong, Kowloon, Hong Kong China; 2 School of Psychology, Fujian Normal University, Fuzhou, China

**Keywords:** Self-objectification, Romantic interpretation bias, Female–male interaction, Relationship contingency, Women, Gender inequality

## Abstract

While various kinds of relationships other than romantic ones exist between men and women in modern society, some individuals tend to simply interpret ambiguous female-male interactions as romantic in nature, which is referred to as romantic interpretation bias (RIB). In this research, we examined the effect of self-objectification on RIB among heterosexual women. Our findings from four studies (*N* = 861), including both cross-sectional surveys (Study 1) and fully controlled experiments (Studies 2, 3A and 3B), consistently showed that women with a higher level of self-objectification exhibited a higher level of RIB when interpreting ambiguous cross-sex interactions. Additionally, this effect could be explained by an increased sense of relationship contingency (Studies 2 and 3B). Importantly, the association between self-objectification and RIB was not only demonstrated in interpreting women’s own ambiguous interactions with men (Studies 1 and 2) but also in interpreting other women’s cross-sex interactions (Studies 3A and 3B). The implications of these findings are discussed.

**Supplementary Information:**

The online version contains supplementary material available at 10.1007/s10508-025-03358-1.

## Introduction

In contemporary society, women frequently interact with men in various roles, including classmates, colleagues, and collaborators. Despite the prevalence of these interactions, a tendency persists among some individuals to misinterpret neutral or ambiguous cross-sex dynamics as indicative of romantic interest, even in the absence of supporting evidence. We define this propensity as romantic interpretation bias (RIB)—the systematic inclination to perceive neutral cross-sex interactions through a romantic lens. While existing literature suggests that women generally underperceive sexual interest from men, research indicates they may not similarly underestimate romantic interest (Koenig et al., 2007). Romance and sexuality are closely linked, and this is particularly the case for women who often contextualize sexual activity within a romantic framework (Meston & Buss, 2007). Although sociocultural constraints frequently discourage the open expression of sexual interest by women (e.g., Clark & Hatfield, 1989), they do not discourage the expression of their romantic interest. Instead, despite the criticisms of feminist scholars, there exist romantic norms that can encourage women to express romantic interest in many societies (e.g., Jackson, 2006; Sharp & Keyton, 2016), such as the widely held belief that life is incomplete without romantic love. Consequently, women may develop a heightened sensitivity to—and a greater willingness to report—perceived romantic cues. This specific tendency to over-attribute romantic intent in cross-sex interactions represents a significant yet under-examined phenomenon in the existing research.

Self-objectification refers to the internalization of sexual objectification that reduces individuals to bodies existing for the pleasure of others (Roberts et al., 2018). Self-objectified people often view themselves as a sexual object, neglecting their own personality and evaluating their worth based on sexual appeal (Fredrickson & Roberts, 1997; Noll & Fredrickson, 1998). Although self-objectification can occur in both men and women, it is primarily observed among women (e.g., Daniels et al., 2020; Wang et al., 2021). Since self-objectified women often adopt a sexualized social script (i.e., men as sexual agents and themselves as sexual objects) to understand cross-sex relationships, and given the strong connection between romance and sex for women, those who display self-objectification may be more likely to exhibit RIB. Additionally, we assume that relationship contingency, the tendency to derive self-worth from romantic relationships, may play a significant role in this process.

### Romantic Interpretation Bias

Related to our proposed RIB, there exists a similar concept known as sexual overperception bias, which refers to the tendency to overestimate the sexual interest of the opposite sex. Since it has been consistently documented that men (but not women) tend to exhibit sexual overperception bias (e.g., Abbey, 1982; Fletcher et al., 2014), studies on this phenomenon have predominantly focused on men. The most widely accepted explanation for the observed sex difference in this bias is rooted in the unique selection pressures faced by men throughout evolutionary history (Abbey, 1982; Lee et al., 2020). According to error-management theory, it is evolutionarily disadvantageous for men (but not women) to miss a mating opportunity, which could lead to the loss of a chance to reproduce with an interested partner of the opposite sex (known as a false miss). As a result, this perception bias may have evolved to help men minimize such costly errors (Haselton & Buss, 2000).

Although women are generally thought to underperceive men’s “sexual” interest, recent research suggests that this phenomenon may not solely be due to sex differences, but rather to individual characteristics like the raters’ sociosexual orientation (Howell et al., 2012; Lee et al., 2020). More importantly, Koenig et al. (2007) suggested that, as sexual overperception bias refers to the misinterpretation of “sexual” interest, it might have confounded short-term (e.g., one-night dating) and long-term relationships (e.g., marriage) to some extent. Therefore, they first explicitly distinguished between sexual and romantic interests and found that females underperceive only their opposite-sex friends’ sexual (but not romantic) interests.

Admittedly, people have the tendency to put romantic relationships together with sexual interaction, although the former may be seen as more complicated in essence than the latter. Based on Sternberg’s Triangular Theory of Love (1986), passion with sexuality is regarded as the motivational element of romantic relationships. It has also been suggested that a romantic relationship cannot exist without sexual desire (Connolly & McIsaac, 2011), especially in light of modern dating culture, where romantic interest is often conflated with sexual interest. In spite of the apparent close association between romance and sex, we believe it should be indeed easier to detect women’s romantic rather than sexual interpretation bias toward the opposite sex due to their different attitudes toward sex and romance. First, women are more likely to make limits on sex but not romantic love as a result of social pressures (e.g., Clark & Hatfield, 1989). Compared to men, women had less permissive attitudes towards sex in casual relationships and exhibited restricted sociosexuality (e.g., Sprecher et al., 2013), suggesting they are often cautious about sex. Unlike sex, however, research has shown that women generally express romantic love and commitment more freely and enthusiastically than men (e.g., Balswick, 1988; Gonzalez & Koestner, 2006). Additionally, it was found that the most dominant reason for women (but not men) to have sex is romance (e.g., expressing love; Meston & Buss, 2007), implying that women may even automatically process sexual encounters as romantic relationships. In fact, studies have shown that romantic relationships play a pivotal role in women’s lives. Women often incorporate intimate relationships into their self-concept (Cross & Madson, 1997; Gabriel & Gardner, 1999). An interview study of college women also highlighted that a significant portion of their time, energy, peer interactions, and conversations was devoted to romantic relationships (Holland, 1992).

Despite the concept differentiation proposed by Koenig et al. (2007) and the evidence showing both differences between sex and romance and the importance of romance for women, most previous studies still focused on males’ sexual overperception bias, and nearly no studies investigated the misinterpretation of romantic interest, particularly among the largely ignored but special population—women. Therefore, in this research, we systematically propose the concept of RIB, which refers to a cognitive bias where neutral and ambiguous interactions between a man and a woman are interpreted as having romantic intent. As with sexual overperception bias, RIB also focuses on biased perceptions or interpretations of cross-gender interactions; however, unlike sexual overperception bias, RIB emphasizes the romance- rather than sex-oriented misinterpretation. Further, we hypothesize that this bias should be especially prominent among women who engage in self-objectification, a point we will explore in greater detail in the following section.

### Women’s Self-Objectification

In contemporary culture, social media platforms often commodify women’s bodies and reinforce societal beauty standards (Bartky, 1997; Bordo, 1993), which promote the objectification of the female body. Women’s self-objectification is a form of internalized control that arises from such objectification (Fredrickson & Roberts, 1997), reducing themselves to instruments of male sexual pleasure while disregarding inner thoughts and feelings (Roberts et al., 2018). Research has demonstrated that self-objectification can lead to a range of detrimental intrapersonal consequences for women, including impaired cognitive performance (Winn & Cornelius, 2020) and significant physical and mental health risks such as eating disorders, depression, and sexual dysfunction (Roberts et al., 2018). Additionally, empirical evidence has underscored the extensive harm linked to self-objectification among women, which can diminish self-efficacy and sense of purpose (Gapinski et al., 2003), hinder personal growth (Wang et al., 2022), promote self-silencing (Saguy et al., 2010), and reinforce justification of gender inequality (Calogero, 2013). Collectively, these negative effects may significantly impact women’s life choices, often confining them to subordinate social roles.

Nevertheless, despite self-objectification being a process that unfolds within interpersonal interactions (i.e., sexual objectification), comparatively few studies have examined its interpersonal consequences for women. Garcia et al. (2016) were among the first to investigate women’s self-objectification in real interpersonal contexts, finding that state self-objectification led to perceptions of decreased comfort and authenticity in interactions with men. This dynamic subsequently impacted women’s relationship agency, career aspirations, and cognitive performance. A more recent study by Guo and Wu (2023) revealed a positive correlation between women's self-objectification and social avoidance, driven by appearance comparisons and diminished self-esteem. Additionally, research has indicated that body surveillance—an index of self-objectification—can positively predict women’s appearance-based rejection sensitivity, defined as a tendency to expect negative evaluations and rejection based on appearance when interacting with others (Park, 2007), due to body shame and a desire for belonging, which in turn can predict increased social anxiety (Teng & Poon, 2020). Building on these findings, our study aims to investigate the relationship between women’s self-objectification and another interpersonal outcome: RIB. Specifically, we were interested in how self-objectification relates to RIB both when women are actively engaging in neutral and ambiguous cross-sex interactions (as the actors) and when they are observing such interactions (as third-party observers).

### Self-Objectification and Romantic Interpretation Bias

The social interaction model of objectification (SIMO), proposed by Gervais et al. (2020), outlines the interactive processes through which women are objectified, positing that objectifying behaviors stem from sexual goals between men and women. It suggests that patriarchal ideology serves as an antecedent phase that may activate women’s self-objectifying sexual goals, thereby influencing their interactions with men. Depending on the degree to which a woman internalizes this ideology, her reactions during interactions with men may vary. Following this framework, self-objectified women who strongly internalize patriarchal beliefs are more likely to conform to the social script that positions men as sexual agents and women as sexual objects during female-male interactions, whether they are participating or observing (Gervais et al., 2020; Kahalon et al., 2018), suggesting they may be more inclined to view such interaction as primarily driven by sexual dynamics. Considering the aforementioned connection between sexual interaction and romance, especially to women, self-objectified women are more likely to perceive the relationship between a man and a woman—even in neutral or ambiguous contexts—as being sex-oriented, which may subsequently develop into a romance-oriented perspective.

In addition, it is helpful to categorize ourselves and others into different social groups so that we can predict each other’s behavior and respond appropriately, and the social category of gender serves as a primary frame for social relations (Ridgeway, 2011). Despite the importance of gender as a category that cannot be ignored, it is often perceived as an implicit background identity that may influence how we expect actors to perform based on their focal identities (e.g., a female teacher should primarily display knowledge in her field, along with warmth and communal concern for her students added by the female identity; Ridgeway, 2011). Self-objectified women, however, may particularly attend to the social category of sex, predicting others’ behavior or motivations primarily or exclusively on the basis of gender, overlooking focal identities. Thus, when interacting with a man or observing the interaction between a man and a woman, it is easier for them to infer that the interaction is dictated by gender roles (e.g., a delicate woman and a powerful man who can protect her) that are closely related to traditional romantic scripts than other social roles (e.g., student and teacher).

In sum, we hypothesized that women with higher levels of self-objectification are more likely to exhibit higher levels of RIB (Hypothesis 1a).

### The Mediating Role of Relationship Contingency

We further predicted that the effect of women’s self-objectification on RIB could be explained by their relationship contingency, also known as relationship-contingent self-worth, which refers to deriving self-worth from romantic relationships (Sanchez et al., 2011). People who hold a strong sense of relationship contingency are likely to believe that if they are not in a relationship, then they are failing at life and are unhappy. Due to the internalization of patriarchal ideology, self-objectified women often accept the structural superiority of men over women (e.g., Calogero, 2013), and frequently perceive themselves as passive objects lacking agency (e.g., Baldissarri & Andrighetto, 2021). As a result, these women tend to rely more on men, the perceived superior party, for attaining resources. Additionally, as forming romantic relationships with men allows them to bond and thus position themselves more favorably, these women may increasingly tie their self-worth to having a romantic partner. In other words, self-objectifying women might exhibit higher levels of relationship-contingent self-worth. Supporting this idea, indirect evidence from a study revealed a positive association between women’s self-objectification and appearance-contingent self-worth (Adams et al., 2017). Not surprisingly, physical attractiveness has been consistently shown to enhance the establishment of romantic relationships, particularly for women (e.g., Buss, 1989; Li & Kenrick, 2006), implying that self-objectified women who base self-esteem on appearance may also base self-esteem on romantic relationships.

Moreover, a heightened sense of relationship contingency may lead to a greater level of RIB when interpreting ambiguous cross-sex interactions. Contingencies of self-worth can drive individuals to set goals and monitor their behaviors to achieve them (Baumeister et al., 1993; Crocker et al., 2003). In this context, relationship contingency can foster a preoccupation with finding a romantic partner and create a sense of mate urgency (Sanchez & Broccoli, 2008). Given the increased risk of missing out on a potential mate in situations of mate urgency, RIB may manifest as an adaptive strategy for biasedly interpreting ambiguous female-male interactions, in line with error-management theory. Consequently, women who are eager to leverage romantic relationships to enhance their self-worth may be more inclined to engage in RIB, as they do not want to miss any opportunities. Additionally, the tendency to interpret neutral and ambiguous cross-sex interactions as romantic can bolster a woman’s self-worth, particularly if she relies heavily on romantic relationships for her self-esteem. This suggests that the emergence of RIB among these women—believing that men interacting with them are expressing romantic interest—may serve to promote their self-esteem to some extent. As well as these internal dynamics, external norms and pressures may also explain the impact of relationship contingency on RIB. Women may be motivated to develop relationship contingency due to enormous societal pressures to find romantic partners (Holland, 1992), which may further contribute to their RIB as they truly expect to find a partner.

Notably, the above-mentioned phenomenon can be further extended from how self-objectified women interpret their own interactions with men to how they interpret other women’s interactions with men due to social projection (i.e., the tendency to assume that others will think and behave similarly to oneself; Krueger, 2000). The social projection theory suggests that such tendency is stronger when individuals judge ingroups than when they judge outgroups (Robbins & Krueger, 2005). Accordingly, if a woman believes engaging in romantic relationships is important for herself, she is likely to assume other women (i.e., ingroup members) more than men (i.e., outgroup members) also hold this belief. As mentioned earlier, self-objectified women tend to incorporate romantic relationships to their self-worth (i.e., show a strong sense of relationship contingency) due to their reliance on men, so they may believe that other women share the same tendency. Moreover, research has shown that women’s self-objectification would impair their cognitive performance (e.g., Quinn et al., 2006; Winn & Cornelius, 2020). Since the tendency of social projection stems from cognitive shortcuts, such as mental simulation or anchoring (Krueger, 1998, 2000), and operates effortlessly and often without conscious awareness (Ames, 2004; Krueger & Clement, 1996), it is highly likely that self-objectified women would exhibit this tendency because they could spend less cognitive effort this way. As evidence, Cheng et al. (2024) revealed that women who engage in self-objectification and have a heightened desire for approval concerning their appearance tend to believe that other women share the same motivations when posting selfies. Combining the theoretical and empirical evidence, we argue that self-objectified women are likely to project their relationship contingency onto other women. They may believe that women, in general, depend on romantic relationships to derive their self-worth and then interpret other women’s neutral and ambiguous interactions with men as romance-oriented.

In conclusion, we hypothesized that relationship contingency could mediate the effect of self-objectification on RIB (Hypothesis 1b). Specifically, self-objectified women often derive their self-worth from romantic relationships, and thus are more likely to interpret their own ambiguous and neutral interactions with men as romance-oriented (Hypothesis 2a). Additionally, they may project their belief onto other women, assuming that other women’s self-worth is similarly tied to romantic relationships, and thus are also more inclined to interpret other women’s ambiguous interactions with men as romantic (Hypothesis 2b).

### The Present Research

This research aimed to explore whether self-objectification would amplify RIB among women (H1a). Additionally, we sought to examine whether increased relationship contingency could mediate this process (H1b). Furthermore, we predicted that the hypothesized effect would be evident not only in self-objectified women interpreting their own ambiguous and neutral interactions with men (H2a) but also in their interpretations of other women’s ambiguous interactions with men (H2b).

To this end, we conducted four studies, including both a cross-sectional survey (Study 1) and fully controlled experiments (Studies 2, 3A, 3B). To ensure the robustness and generalizability of our findings, we applied various manipulation methods for self-objectification, developed different measures of RIB, and adopted samples from different cultures (i.e., China, the UK, and the US). In addition, RIB was considered from not only the first-person perspective (i.e., participants as actors in a female-male interaction; Studies 1 and 2) but also the third-person perspective (i.e., participants as third-party observers; Studies 3A and 3B). All study procedures were approved by the Institutional Review Board of the corresponding author’s institution, and informed consent was obtained from each participant. Given the cultural diversity and cross-national nature of the studies, we paid special attention to adapting our materials in different countries.^1^ Data and study materials can be accessed via https://osf.io/mysf8/overview?view_only=87761784d893435e82b26d0c22781789.

### Sample Size Determination

According to Monte Carlo simulations (Schönbrodt & Perugini, 2013), we aimed to recruit 250 participants in order to obtain stable estimates of bivariate correlations (Study 1). As a rule of thumb, experiments require at least 50 participants per condition (Faul et al., 2007). For our experimental studies (Studies 2-3B), we predetermined a sample size of 100 participants per condition based on the standards adopted in recent research (e.g., Chen et al., 2024; Wang et al., 2023a, 2023b). Sensitivity power analyses (Faul et al., 2009) showed that minimum effect sizes of *f* = .20 (Study 2, *N* = 196), *f* = .19 (Study 3A, *N* = 221), and *f* = .20 (Study 3B, *N* = 194) could be detected under standard criteria (α = .05, 80% power).

### Study 1

The purpose of Study 1 is to provide preliminary support for H1a by measuring women’s trait self-objectification and RIB from a first-person perspective. We predicted that women with higher levels of trait self-objectification tend to interpret their own ambiguous interactions with men as romance-oriented. We used two scales to comprehensively capture self-objectification in Study 1, aiming to rule out the possibility that the effect is not observable when a certain measure is used.

## Method

### Participants

A total of 250 Chinese heterosexual women (*M*_age_ = 32.33 years, *SD* = 7.65) who correctly answered the attention check items were recruited via Credamo, an online survey platform in China comparable to Prolific. There were 4.0% of participants who had never been in a romantic relationship, 11.6% who had previously been in one but were not currently in one, and 84.4% who were currently in a romantic relationship.

### Procedure and Measures

Upon providing informed consent, participants first provided their demographic information (including age, sexual orientation, current relationship status, height, and weight^2^). Then, they responded to two different scales measuring trait self-objectification in a random order, followed by the measure of RIB. Finally, they answered several scales of control variables.

#### Self-Objectification

To ensure the robustness of the results, two measures of trait self-objectification, i.e., Self-Objectification Questionnaire (SOQ; Noll & Fredrickson, 1998) and Self-Objectification Beliefs and Behaviors Scale (SOBBS; Lindner & Tantleff-Dunn, 2017), were adopted. SOQ asks participants to rank the importance of ten attributes related to physical self-concept—five appearance aspects and five competence-related aspects of the body—from 0 (least important) to 9 (most important). SOQ scores were computed by subtracting the sum of rankings of competence-based attributes from those of appearance-based attributes. The possible range of scores was − 25 to + 25, with higher scores indicating greater trait self-objectification. SOBBS is another widely used scale for measuring trait self-objectification and consists of two factors: the observer’s perspective (i.e., thinking the body as an observer would) and the body as self (i.e., treating the body as if it is capable of representing the self as a person). Participants rated their agreement with 14 items on a scale from 1 (*strongly disagree*) to 5 (*strongly agree*). Sample items include “I have thoughts about how my body looks to others even when I am alone” (the observer’s perspective; α = .94) and “How I look is more important to me than how I think or feel” (the body as self; α = .93). Following previous studies (e.g., Ozimek et al., 2023), when testing the hypotheses, scores of all 14 items were averaged, with higher scores indicating greater levels of trait self-objectification (α = .96).

#### Romantic Interpretation Bias (First-Person)^3^

The RIB was measured using two hypothetical scenarios depicting ambiguous interactions between the participant and a male character. The first scenario describes a situation that occurs in a volunteering organization. The participant was asked to imagine that in this organization, she was planning a cultural festival with a male character named Xiao Chen (XC). The two often discussed the upcoming festival together, especially the section concerning music performances. One day, after a discussion, XC sent her a message mentioning that a live band would be performing this Saturday whose music was very similar to what they had been discussing and asking whether she would be interested in attending together. In the second scenario, a similar situation occurs in a cooking class. The participant was asked to imagine that she recently took a cooking class in which a male member named Xiao Ling (XL) participated. Over the past few weeks, she has collaborated and cooked several times with XL, and they have discovered some common interests in cooking. One day, after class, XL sent her a message stating that he had found a food festival this weekend with some of the chefs and food they had discussed and asking if she would like to participate together. The presentation of the two scenarios was counterbalanced.

In response to each scenario, participants expressed their agreement with five items regarding the extent to which they viewed the interaction as romantic, ranging from 1 (*strongly disagree*) to 7 (*strongly agree*). Sample items include “XC/XL’s behavior seems to imply a desire for a romantic connection” and, “I think XC/XL views our interactions as just friendly (reverse-coded).” The RIB score of each scenario was calculated by averaging the scores of the five items (α = .96 for both scenarios; *r* = .67, *p* < .001). The overall RIB score was then calculated by averaging the RIB scores of the two scenarios, with higher scores indicating a greater tendency to interpret the ambiguous female-male interaction as romantic.

#### Control Variables

This study controlled for participants’ relationship status, self-esteem, social desirability, self-perceived attractiveness, and BMI. As revealed by Sanchez and Broccoli (2008), single women exhibited a greater degree of self-objectification following relationship priming, whereas women in relationships exhibited a decreased level of self-objectification. Since the RIB measure may somewhat prime participants with romantic relationships, we controlled for relationship status for more accurate results. As self-esteem is typically associated with self-objectification (Tolman et al., 2006), we controlled for it to ensure that our results were not affected by the level of self-esteem. To ensure that our results remained unchanged if we accounted for participants’ impression management, especially considering that our study relates to romantic situations where impression management is quite common (Sanchez & Broccoli, 2008), we controlled for social desirability. Self-perceived attractiveness was controlled since it was shown to be positively correlated with romantic self-confidence (Bale & Archer, 2013) and sexual overperception bias (Samara et al., 2021), which might be related to RIB. BMI was controlled since it is positively related to appearance anxiety and negatively related to body esteem (Tiggemann & Lynch, 2001), both of which have the potential to influence notions of self-objectification and romantic beliefs.

#### Self-Esteem

Self-esteem was measured using the 10-item Rosenberg Self-Esteem Scale (Rosenberg, 1965). A sample item is “I feel that I have a number of good qualities” (1 = *strongly disagree*, 4 = *strongly agree*). Higher average scores on the 10 items indicate greater self-esteem (α = .81).

#### Social Desirability

Social desirability was measured using the Lie subscale of the short-scale Eysenck Personality Questionnaire (Eysenck & Eysenck, 1975). For the brevity of the survey, only three items were selected based on the highest factor loadings found in a recent study conducted among Chinese participants (Dong et al., 2024), including “Were you ever greedy by helping yourself to more than your share of anything?”, “Are all your habits good and desirable ones?”, and “Have you ever said anything bad or nasty about anyone?” This scale uses a dichotomous (Yes/No) response format. Total scores were calculated by summing responses, with higher scores indicating higher levels of social desirability (α = .76).

#### Self-Perceived Attractiveness

Self-perceived attractiveness was measured with three items adopted from Teng et al. (2022). Participants indicated on a scale from 1 (*very unattractive/very unsatisfied*) to 9 (*very attractive/very satisfied*) the extent to which they agreed with the following questions: “How is your physical attractiveness compared to people around you?”, “How do you rate your physical appearance?”, and “How satisfied are you with your physical appearance?”. Scores were averaged, with higher scores corresponding to greater levels of self-perceived attractiveness (α = .92).

## Results and Discussion

As hypothesized, RIB was positively correlated with self-objectification: SOQ, *r*(250) = .39, *p* < .001, and SOBBS, *r*(250) = .44, *p* < .001 (see Table 1), suggesting that women with higher levels of trait self-objectification tend to construe ambiguous female-male interactions involving themselves as more romance-oriented.

**Table 1:** Descriptive statistics and bivariate correlations among key variables in Study 1 (*N* = 250)

	SOQ	SOBBS	RIB	Relationship status	Self-esteem	Social desirability	Self-perceived attractiveness	BMI
SOQ	–							
SOBBS	.67^***^	–						
RIB	.39^***^	.44^***^	–					
Relationship status	.13^*^	.08	.03	–				
Self-esteem	.01	–.05	–.06	.45^***^	–			
Social desirability	–.04	–.12	–.23^***^	.24^***^	.21^***^	–		
Self-perceived attractiveness	.21^***^	.17^**^	.003	.43^***^	.65^***^	.23^***^	–	
BMI	.07	–.06	.13^*^	.19^**^	.15^*^	.03	.02	–
*Mean*	–5.14	3.01	3.37	–	3.32	1.58	6.42	20.38
*SD*	14.13	1.06	1.37	–	0.40	1.20	1.38	2.16

*SOQ* Self-Objectification Questionnaire, *SOBBS* Self-Objectification Beliefs and Behaviors Scale

^*^p < .05, ^**^p < .01, ^***^p < .001

For relationship status

1 = I have never been in a romantic relationship, 2 = I have been in a romantic relationship before but am currently not in one, 3 = I am currently in a romantic relationship

Hierarchical linear regression analyses were then conducted to examine the relationship between trait self-objectification and the RIB with control variables included. There is no severe multicollinearity among the variables (see Supplementary Materials for relevant results). Both SOQ and SOBBS were still significantly associated with RIB after the above-mentioned variables were controlled: *b* = 0.04, *SE* = 0.01, *t* = 6.30, *p* < .001 (SOQ; see Table 2); *b* = 0.55, *SE* = 0.08, *t* = 7.29, *p* < .001 (SOBBS; see Table 3), indicating that the positive correlation between women’s self-objectification and RIB was not influenced by their relationship status or different levels of self-esteem, social desirability, self-perceived attractiveness, and BMI. Notably, these results remained unchanged when the control variables were not included in the models: *b* = 0.04, *SE* = 0.39, *t* = 6.74, *p* < .001 (SOQ); *b* = 0.57, *SE* = 0.07, *t* = 7.64, *p* < .001 (SOBBS). Overall, the results of Study 1 supported H1a.

**Table 2:** Results of the hierarchical linear regression analysis in Study 1 (SOQ as the predictor)

Variables	Model 1			Model 2		
	*b*	*SE*	*p*	*b*	*SE*	*p*
(constant)	2.27	1.00	.024	2.82	.93	.003
Relationship status	0.20	.20	.315	0.10	.19	.591
Self-esteem	− 0.48	.29	.100	− 0.15	.27	.575
Social desirability	–0.29	.07	< .001	− 0.24	.07	< .001
Self-perceived attractiveness	0.11	.08	.171	− 0.02	.08	.841
BMI	0.09	.04	.024	0.07	.04	.056
SOQ				0.04	.01	< .001
*F*	4.623			11.083		
*df*_1_*, df*_2_	5, 244			6, 243		
*R*^2^	.087			.215		
Δ*R*^2^				.128^***^		

*SOQ* Self-Objectification Questionnaire

^***^p < .001

**Table 3:** Results of the hierarchical linear regression analysis in Study 1 (SOBBS as the predictor)

Variables	Model 1			Model 2		
	*b*	*SE*	*p*	*b*	*SE*	*p*
(constant)	2.27	1.00	.024	0.18	.95	.847
Relationship status	0.20	.20	.315	0.09	.18	.625
Self-esteem	− 0.48	.29	.100	− 0.08	.27	.771
Social desirability	− 0.29	.07	< .001	− 0.21	.07	.002
Self-perceived attractiveness	0.11	.08	.171	− 0.03	.08	.711
BMI	0.09	.04	.024	0.10	.04	.006
SOBBS				0.55	.08	< .001
*F*	4.623			13.519		
*df*_1_*, df*_2_	5, 244			6, 243		
*R*^2^	.087			.250		
Δ*R*^2^				.164^***^		

*SOBBS* Self-Objectification Beliefs and Behaviors Scale

^***^p < .001

### Study 2

Study 2 aimed to manipulate women’s state self-objectification and examine its causal effect on RIB to further test H1a. In this study, RIB was assessed using a different measure that was more ambiguous and less susceptible to interference. Of equal importance, the mediating effect of relationship contingency (H1b), specifically from the first-person perspective (H2a), was also explored.

## Method

### Participants and Design

We recruited 196 Chinese heterosexual women who passed the attention check through Credamo (*M*_age_ = 30.34 years, *SD* = 6.70). During the data checking stage, their written responses were carefully read and analyzed with the assistance of AI detection tools to ensure that they were human respondents. Among the participants, 9.2% had never been in a romantic relationship, 12.8% had been in one previously but were not currently involved, and 78.1% were currently involved. All participants were randomly assigned to either the self-objectification (*N* = 97) or the self-perception condition (*N* = 99).

### Procedure and Materials

To manipulate state self-objectification, a passage reading along with a recalling task was utilized. Participants assigned to the self-objectification group were required to read a short passage in which they were informed that, influenced by cultures, media as well as interpersonal interactions, women sometimes internalize the gaze on their appearances and thus would have experiences like looking in the mirror and asking themselves how they look that day, or thinking about looking good rather than feeling comfortable when buying clothes. In other words, women could emphasize their physical appearances rather than inner qualities (such as personalities). After that, they were asked to recall and write about their similar experiences. In contrast, participants assigned to the self-perception group first read a passage about the fact that people can sometimes understand their internal states via external behaviors and thus would have experiences like recognizing the preferred time for creative work by being able to do more tasks effectively at a given time or knowing the attitude toward a certain cuisine because of always choosing a certain flavor of food. Then, they were asked to recall and write down personal experiences that could reflect their self-perceptions.

Following the manipulation, participants responded to three items assessing their state self-objectification on a scale from 1 (*strongly disagree*) to 7 (*strongly agree*), including “Right now, I am thinking about how my body looks to others”, “Right now, I am imagining what my body looks like to others”, and “Right now, I feel that my body is what gives me value to other people” (α = .76; Cheng et al., 2024).

Then, relationship contingency was measured with four items developed by Sanchez and Kwang (2007), adapting the specific reference of “significant other” from “boyfriend or girlfriend” to “boyfriend or husband” as we only focused on women in this study. The items included: “When I do not have a significant other (i.e., boyfriend or husband), I feel badly about myself”, “I feel worthwhile when I have a significant other (i.e., boyfriend or husband)”, “When I have a significant other (i.e., boyfriend or husband), my self-esteem increases”, “My self-esteem depends on whether or not I have a significant other (i.e., boyfriend or husband)” (1 = *strongly disagree*, 7 = *strongly agree*; α = .84). Higher average scores represent higher levels of relationship contingency.

**Fig. 1:**
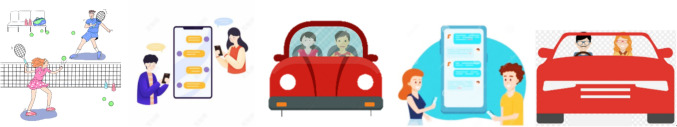
Cartoon pictures depicting ambiguous female-male interactions used in Study 2 (*Pictures 1, 2, 3*) as well as Study 3B (*Pictures 1, 4, 5*)

After that, we assessed the RIB from the first-person perspective with a newly designed picture measure. The picture chosen deliberately depicted ambiguous female-male interactions, avoiding the interference that may arise from the verbal descriptions of the scenarios. Participants were sequentially (each on a separate page) presented with three cartoon pictures depicting different female-male interactant situations (i.e., playing tennis, chatting online, and riding a car together) in random order (see Fig. 1).^4^ As each picture was presented, the participant was asked to imagine that she was the female protagonist in the picture and was doing the activities depicted with a male companion. Each picture was accompanied by three items designed to assess RIB, including “How likely do you think it is that the man you see considers his relationship with you to be pure friendship? (*reverse-coded*)” (1 = *very much unlikely*, 7 = *very much likely*), “To what extent do you think the interaction between the man and you in this situation looks like a potential date?” (1 = *not at all*, 7 = *very much so*), and “In your opinion, what type of relationship do you think the man desires with you in this situation?” (1 = *just friends*, 9 = *romantic partners*). The three items showed a good degree of internal consistency in all three picture measures: α = .90 (playing tennis); α = .83 (chatting online); α = .85 (riding a car). Our first step in developing the RIB score was to average the scores on each item across different picture measures. Given that the three items were measured on different scales (the first two were scored on a seven-point scale, and the third was scored on a nine-point scale), we then standardized the three mean variables. As the final step, we averaged the three standardized variables to obtain an overall RIB score, with higher scores indicating a stronger RIB.

Finally, the same control variables and demographic information as in Study 1 were collected before they were thanked and debriefed.

## Results and Discussion

A significant difference in state self-objectification was obtained between participants in the self-objectification (*M* = 5.35, *SD* = 1.09) and the self-perception (*M* = 4.68, *SD* = 1.09) groups, *t*(194) = 4.27, *p* < .001, Cohen’s *d* = 0.61, validating the effectiveness of our manipulation.

Women in the self-objectification group exhibited a higher level of RIB when interpreting ambiguous female-male interactant situations than those in the self-perception group: *M* = 0.16, *SD* = 0.91 vs. *M* = –0.16, *SD* = 0.85; *t*(194) = 2.58, *p* = .011, Cohen’s *d* = 0.37. Those in the self-objectification (vs. self-perception) group also showed a higher level of relationship contingency: *M* = 4.04, *SD* = 1.41 vs. *M* = 3.61, *SD* = 1.39; *t*(194) = 2.14, *p* = .034, Cohen’s *d* = 0.31. These effects remained significant after controlling for the same control variables as in Study 1: *F*(1,189) = 7.11, *p* = .008, η_p_^2^ = .036 (RIB); *F*(1,189) = 5.60, *p* = .019, η_p_^2^ = .029 (relationship contingency). These results suggest that women who were guided to focus on their physical appearance rather than inner feelings were more likely to perceive ambiguous cross-sex interactions involving themselves as romantic and derive self-worth from romantic relationships, providing causal evidence for H1a.

**Fig. 2:**
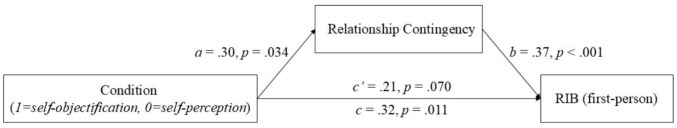
The mediation model in Study 2

The mediation analysis (a bootstrap analysis of 5,000 random samples; Preacher & Hayes, 2008) indicated that relationship contingency (standardized) could mediate the causal relationship between the condition (self-objectification vs. self-perception) and RIB, a*b = .11, *SE* = 0.06, 95% CI = [0.013, 0.229] (see Fig. 2). Women who were instructed to focus on their physical appearance rather than inner feelings tended to derive their self-worth from romantic relationships, thereby displaying a greater tendency to perceive ambiguous cross-sex interactions involving themselves as romantic, which initially supported H2a.

### Study 3

The primary aim of Study 3 was to test whether the causal effect of self-objectification on RIB found in Study 2 can be extended to interpreting third-person interactions. That is, we are interested in exploring whether the RIB seen in interpreting a woman’s own interaction with a man can further be projected onto other irrelevant third parties and observed in interpreting other women’s ambiguous interactions with men.

### Study 3A

Study 3A utilized a different method to manipulate state self-objectification to test its causal effect on the RIB in interpreting third-person female-male interactions (H1a).

## Method

### Participants and Design

We recruited 221 Chinese heterosexual women who correctly answered the attention check questions through Credamo (*M*_age_ = 31.00 years, *SD* = 8.07). As in Study 2, their written responses were carefully read and analyzed with the assistance of AI detection tools to ensure that they were human respondents. Seven percent of participants had never been involved in a romantic relationship, 16.3% had been involved in one previously but were not currently involved, and 75.0% were currently involved. Participants were randomly assigned to either the self-objectification (*N* = 112) or the control condition (*N* = 109).

### Procedure and Materials

A writing task adapted from Register et al. (2015) was used to manipulate the temporary state of self-objectification. Those assigned to the self-objectification group were asked to take a moment to look at themselves from someone else’s perspective and try to write about how other people would see and evaluate their physical appearance. In contrast, those assigned to the control group were asked to take a moment to think about the activities they participated in the last 24 h and try to write down these activities in chronological order.^5^

We then used the same two scenarios as in Study 1 to measure RIB but modified them for interactions between hypothetical women and men from the third-person perspective. That is, instead of asking participants to imagine that they were in a volunteering organization or cooking class with the male character XC or XL, we asked them to imagine that female characters Xiao Han (XH) and Xiao Man (XM) were engaged in the situations with XC and XL, respectively. Upon reading each scenario, participants responded to the same five items as in Study 1, albeit from the third-person perspective (α = .94 for both scenarios; *r* = .51, *p* < .001).

Finally, participants reported the same control variables and demographic information as in Study 1 before they were thanked and debriefed.

## Results and Discussion

Participants in the self-objectification condition showed a stronger RIB when observing and interpreting other female-male interactions from a third-person perspective than those in the control condition: *M* = 4.02, *SD* = 1.17 vs. *M* = 3.51, *SD* = 1.16; *t*(219) = 3.20, *p* = .002, Cohen’s *d* = 0.43. This effect remained significant after controlling for the same control variables as in previous studies, *F*(1, 214) = 5.28, *p* = .023, η_p_^2^ = .024. These results indicate that women primed with self-objectification were more likely to interpret other women’s ambiguous interactions with men as romance-oriented, successfully supporting H1a from a third-person perspective.

### Study 3B

Study 3B served two main aims. First, in addition to testing again the focal causal relationship found in Study 3A, this study further examined whether relationship contingency projected on all women could account for this causal relationship (H2b). Second, we aimed to test the generalizability of the hypothesized effect using a sample from different cultures.

## Method

### Participants and Design

A total of 194 heterosexual women from the UK and the US who passed the attention check were recruited through Prolific (*M*_age_ = 40.03 years, *SD* = 12.11; 66.5% White, 11.9% Black, 12.9% Asian, 8.8% Mixed or others). As in Study 2, their written responses were carefully read and analyzed with the assistance of AI detection tools to ensure that they were human respondents. There are 4.6% of participants who had never been involved in a romantic relationship, 19.6% who had been involved in one in the past but were not currently involved, and 75.8% who were currently involved. Participants were randomly assigned to either the self-objectification or the control group, resulting in 97 participants per condition.

### Procedure and Materials

The same tasks as in Study 2 were used to manipulate state self-objectification. Rather than using self-perception as the contrasting group, a more neutral control group was used in this study to better ensure the effect of manipulation on RIB was due to increased levels of self-objectification rather than decreased levels of self-perception. In particular, participants in the control group were asked to first read that various places serve different functions to satisfy people’s daily needs in a short passage and then recall and write down experiences during their last visit to one place (e.g., bookstores, supermarkets). The same three items as in Study 2 (α = .90) were adopted to check the effectiveness of the manipulation.

Then, the same four items as in Study 2 were slightly modified to measure relationship contingency projected on women as a whole. A sample item is “A woman might feel worthwhile when she has a significant other (i.e., boyfriend or husband)” (α = .86).

Next, RIB was assessed using the same picture measures as in Study 2 with two minor revisions. First, the instructions were changed to a third-person perspective, asking participants to imagine seeing a woman and a man engaging in the activities depicted in each picture one day and then answering questions based on their understanding. Second, two of the pictures used in Study 2 were replaced with two new ones depicting the same interactant situations with characters more resembling Westerners to avoid noises and biased perception associated with outgroup members (see Fig. 1). The same three items assessing RIB as in Study 2 still showed a good degree of internal consistency in all three picture measures: α = .87 (playing tennis); α = .85 (chatting online); α = .80 (riding a car).

Finally, participants provided answers to the same control variables and demographic information as in Study 1 before they were thanked and debriefed.

## Results and Discussion

A significant difference in state-level self-objectification between participants in the self-objectification (*M* = 4.00, *SD* = 1.68) and the control (*M* = 3.32, *SD* = 1.78) conditions emerged, *t*(192) = 2.71, *p* = .007, Cohen’s *d* = 0.39, validating our manipulation method.

As predicted, women in the self-objectification condition showed an increased level of RIB than those who were in the control condition: *M* = 0.15, *SD* = 0.82 vs. *M* = –0.15, *SD* = 0.95; *t*(192) = 2.30, *p* = .023, Cohen’s *d* = 0.33. Additionally, those in the self-objectification (vs. control) condition showed a stronger tendency to project relationship contingency on other women: *M* = 4.52, *SD* = 1.36 vs. *M* = 4.16, *SD* = 1.17; *t*(192) = 2.00, *p* = .047, Cohen’s *d* = 0.29. After controlling for the same variables as in previous studies, these effects remained significant: *F*(1,177) = 5.10, *p* = .025, η_p_^2^ = .028 (RIB); *F*(1,177) = 4.37, *p* = .038, η_p_^2^ = .024 (projected relationship contingency). These results show that women primed with a self-objectified mindset were more inclined to interpret ambiguous female-male interactions they observe as romance-oriented and believe that women in general derive their self-worth from romantic relationships, further supporting H1a.

**Fig. 3:**
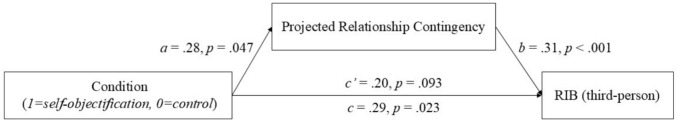
The mediation model in Study 3B

Finally, the similar mediation analysis as in Study 2 showed that projected relationship contingency (standardized) could significantly mediate the causal relationship between the self-objectification group and RIB from the third-person perspective, a*b = .09, *SE* = 0.05, 95% CI = [0.003, 0.197] (see Fig. 3). Women primed with a self-objectified mindset believed that women in general derive their self-worth from romantic relationships, resulting in a greater tendency to perceive other women’s neutral interactions with men as romantic, which further supported H1b and H2b.

## General Discussion

Four studies involving different designs and measurements supported our hypotheses that self-objectified women tend to interpret cross-sex interactions as romance-oriented (i.e., RIB; H1a), which can be explained by relationship contingency (H1b). Importantly, this phenomenon can be observed both in self-objectified women’s interpretations of their own interactions with men and in their interpretations of other women’s interactions with men (H2 a and b). The results have been demonstrated in several cultural contexts, including China, the UK, and the US, indicating that the phenomenon is somewhat generalizable. Study 1 first revealed the positive association between trait self-objectification and RIB in interpreting the first-person interaction with a cross-sectional survey. Then, Study 2 demonstrated that temporarily introducing self-objectification would enhance first-person RIB via an increased sense of relationship contingency. Studies 3A and 3B, employing samples from different cultures, further showed that the causal effect of self-objectification on RIB could be extended to women’s interpretation of cross-sex interactions from the perspective of a third party. Moreover, Study 3B demonstrated that this effect could be accounted for by relationship contingency projected on all women.

### Theoretical Contributions

#### Self-Objectification

This research is among the first to show the impact of self-objectification on women’s interpretation of their interpersonal experiences, providing empirical evidence for SIMO, which suggested that within the cultural context of patriarchy, romantic interactions often involve the objectification of women, following the social script of men acting as sex agents and women as sex objects (Gervais et al., 2020). Our findings support this framework by showing that women who self-objectify are indeed more likely to adopt such social scripts, thereby interpreting ambiguous female-male interactions as romantic in nature.

This study expands our knowledge of women’s self-objectification by revealing a new possibility of why self-objectified women often feel lonely. Teng et al. (2019) has preliminarily showed the positive association between self-objectification and loneliness and that increased body shame and decreased general self-esteem could account for this relationship. Moreover, Dvir et al. (2021) further suggested that mere sexual objectification can result in feelings of ostracism because the perpetrator is ignoring the target’s personality and focusing instead on their physical appearance. Our results offer a new perspective for understanding women who self-objectify or experience sexual objectification’s feelings of loneliness: in overly construing female-male interactions as romantic, these women may end up believing that women and men cannot form Platonic friendships, thereby losing many opportunities to cultivate interpersonal relationships.

Additionally, this study contributes empirical evidence to previous assumptions that self-objectified women have a diminished sense of agency in their lives. Our research reveals that self-objectified women tend to place a strong emphasis on engaging in romantic relationships with men, implying their inclination to accept romantic norms or heteronormativity. Feminist scholars have criticized these societal norms because they often reinforce gender inequality and limit women’s agency (e.g., Jackson, 2006). Thus, the endorsement of these norms by self-objectified women may explain Fredrickson and Roberts’ (1997) claim that self-objectification reduces women’s agency.

#### Romantic Interpretation Bias and Sexual Overperception Bias

This study also broadens the scope of research on sexual overperception bias from several aspects. First, although Koenig et al. (2007) have distinguished between perceptions of sexual and romantic interest, our study is the first to systematically investigate the influential factor of misinterpretation of romantic interest to the best of our knowledge. In addition, Koenig et al. (2007) focused on misperceptions of romantic interest in opposite-sex friendships that often include sexual as well as romantic attraction toward each other in nature (Reeder, 2000; Rose, 1985), whereas our research focused on more general interactions between men and women regardless of their actual relationship. In other words, we focused more on neutral instead of romantic-permissive contexts, making it easier to generalize the results. Also, notably, although a series of studies have shown that the actor’s tendency to overperceive sexual interest of the opposite sex may be projected on the interacted opposite sex (e.g., Koenig et al., 2007; Lee et al., 2020; Samara et al., 2021), our research first demonstrated that this tendency can also be projected on other members of the actor’s ingroup, resulting in overperceiving romantic signals in irrelevant cross-sex interactions.

As romance has always been considered important in facilitating sex (Boislard et al., 2016), our research further suggested that previously observed sex differences in sexual overperception bias may partially be explained by the sex-oriented approach used. In these studies, participants were often asked to report whether the opposite sex was friendly or “flirtatious/seductive” (e.g., Abbey, 1982; Howell et al., 2012) or directly assess whether the opposite sex has wrongly inferred their “sexual” interest (e.g., Haselton, 2003). In accordance with error management theory, it is more costly for women (but not men) to engage in sex with an uncommitted opponent of the opposite sex (Haselton & Buss, 2000; Haselton & Nettle, 2006). As well, empirical research found that most women are uncomfortable discussing sex and fear judgment for talking about sexual desire (e.g., Montemurro et al., 2015). Thus, the observed effect of women underperceiving the sexual interest of the opposite sex may be merely an effect of “underreporting” due to the need to protect (sexual) reputation. If this is the case, extending the sex-oriented bias (i.e., sexual overperception bias) to romance-oriented one that is less subject to criticism (i.e., RIB) may provide an opportunity to reach a deeper understanding of the cognitive bias in interpreting cross-sex interactions, especially for special populations like women.

### Practical Implications

As discussed previously, self-objectification may reduce the agency of women by causing them to focus heavily on romantic interest from men in social interactions. Considering that the promotion of women’s agency is important for achieving gender equality (e.g., Bayeh, 2016), it is necessary to focus on the sociocultural root of self-objectification and to intervene against it. From the perspective of body politics, our bodies are shaped by broader social and cultural factors (Brown & Gershon, 2017). Exposure to cultural norms (e.g., those reflected in social media) that frequently depict “ideal” standards of women’s body may cause them to excessively focus on appearances within patriarchal society (Bordo, 1993), thereby experiencing self-objectification. In other words, our findings suggest that women’s agency need to be protected from narrowly oriented and oppressive societal norms, such as those pertaining to thinness standards (Bartky, 1997; Bordo, 1993).

In addition, as underscored by the global *#MeToo* movement that has brought much attention to the prevalence of sexual harassment and assault, women are frequently subjected to sexual violence in a variety of forms. Thus, finding solutions to such issues is imperative. In empirical research, sexual objectification has been identified as a potential factor contributing to women’s sexual victimization (e.g., Gervais et al., 2014; Haikalis et al., 2017). More importantly, this link was found to be mediated by increased self-objectification (Franz et al., 2016), indicating that women who self-objectify are more likely to experience sexual assault. Meanwhile, it has long been recognized that sexual overperception bias plays a significant role in predicting men’s intentions to engage in sexual aggression (e.g., Bondurant & Donat, 1999; Bouffard & Miller, 2014). It has been observed that male participants tended not to label nonconsensual intercourse initiated by a male character as sexual assault when reading that the female character involved showed some interest in interacting with the male character (Yndo & Zawacki, 2020). Even though lacking direct evidence from the female perspective, one can reasonably assume that women who overperceive romantic intent from men when engaging in cross-sex interactions may be less likely to recognize a sexual assault based on evidence derived from the male perspective, making them more likely to suffer sexual aggression. This assumption may somewhat explain why self-objectified women seem more vulnerable to sexual attack, highlighting the importance of addressing structural and systemic factors that shape women’s self-objectification and thus their misinterpretations of cross-sex interactions. For example, by launching programs focusing on challenging societal narratives that normalize objectification and reinforce unequal power dynamics between genders, we may possibly reduce incidents of sexual assault.

### Limitations and Future Directions

Firstly, in future research, RIB may be examined as a mechanism for explaining the previously reported effect that women’s self-objectification impairs their cognitive performance (e.g., Quinn et al., 2006; Winn & Cornelius, 2020). In these studies, an excessive focus on appearance was used as the primary explanation for this impairment (e.g., Quinn et al., 2006). Since RIB is basically a cognitive bias that indicates an excessive emphasis on romantic signals between different sexes when engaging in interpersonal interactions, our results suggest that self-objectified women may also experience cognitive deficits as a result of RIB. Moreover, prior studies found that women’s strong self-objectification may contribute to their poor performance in many areas, such as academic achievement (Dwivedi et al., 2022). Wherever in academic settings or workplace, interpersonal interactions between men and women are unavoidable. Therefore, considering romantic possibilities with every man interacted may occupy plenty of time and cognitive resources of self-objectified women, which may explain their poor performance reported in previous studies.

Furthermore, all studies were conducted via online platforms and the experimental tasks were based on imagined scenarios rather than interactions in real-life situations (e.g., actual “dates”). Although some methods have been implemented to ensure the quality of the data, it is difficult to guarantee that every participant is a real human who is paying sufficient attention to answer the questionnaire. Besides, although the use of hypothetical measures can reduce interference from social desirability and our supplementary study confirms to some degree the ecological validity of these measures, it has been recognized that individuals may experience different decision-making processes or even brain activity when facing hypothetical versus real-life choices (e.g., Camerer & Mobbs, 2017). Consequently, future research can recruit participants in the lab for face-to-face interactions to further investigate the effect revealed in our study.

Finally, our research focused primarily on identifying some commonly observed effects but did not consider cultural differences in them, which could be an important factor influencing outcomes since people in different cultural contexts may have different views on interpersonal relationships (e.g., Lou & Li, 2017). For example, relational mobility, namely the degree to which a given society offers individuals the opportunity to form new relationships (Yuki & Schug, 2012), might affect the strength of the focal association. A society with a greater degree of relational mobility provides a larger degree of freedom for individuals to choose new relationships in accordance with their personal preferences, while one with a lower degree of relational mobility tends to create relatively stable relationships that are dependent on one’s close-knit environment (Lou & Li, 2017). Consequently, the lack of relational mobility in a given culture may impair women’s imagination of interpersonal interactions with others, including men, thus strengthening the effect of self-objectification on RIB. Moreover, future research may also investigate the impact of women’s romantic relationship status on the outcomes. For example, women who are currently in relationships may possess higher levels of romantic confidence than single women, resulting in a greater tendency to assume other men have romantic interests in them as well. In light of these possibilities, it is important to take an intersectionality perspective in future studies to more systematically examine potential differences shaped by diverse aspects of one’s identity (Gill, 2009).

### Conclusions

In summary, the current research shows that women with strong self-objectification tend to interpret ambiguous and neutral female-male interactions as romantic, whether they are actively engaged in the interaction or observing it from a third-party perspective. In addition, relationship contingency can explain this effect, either embedded within self-objectified women themselves or projected upon other women. These findings identify self-objectification as a potential risk factor influencing the quality of women’s interpersonal relationships, highlighting the need to reduce self-objectification among them. Educators can integrate lessons that foster critical thinking and self-reflection and deconstruct dating norms to help self-objectified female students to build healthier relationship expectations. Clinicians, on the other hand, can adopt a new perspective in therapy by exploring how self-objectification affects women’s self-worth sources and interpretations of cross-sex interactions, thereby offering better support for women with interpersonal problems. Our findings also reveal the potential impact of compulsory heterosexuality on women, indicating that relevant norms and ideals can have a significant influence on women’s bodies and interpersonal expectations (Ahmed, 2013). Consequently, policymakers can launch media literacy programs that address heteroromantic scripts, promote anti-normative activities as well as reduce societal signs of heteronormativity to assist in dismantling heterosexual romantic hegemony. By implementing these strategies, we may foster more respectful cross-gender interactions and contribute to advancing social equality.

## Supplementary Information

Below is the link to the electronic supplementary material.

Supplementary file1 (DOCX 20 KB)

## Data Availability

The data and study materials can be accessed via https://osf.io/mysf8/overview?view_only=87761784d893435e82b26d0c22781789

## Footnotes

1 Before conducting Study 3B, which employed samples from the UK and the US, we sought informal feedback from several individuals representing both cultures to ensure translation clarity and cultural relevance of our materials. According to their feedback, the materials were not misunderstood or encountered any major issues, suggesting that the adaptations made were appropriate to the respective cultural contexts.

2 Height and weight were collected to calculate BMI, a measure of body mass, to be included as a control variable in data analysis.

3 The ecological validity of both RIB scenario and picture measures was confirmed after the data collection of this research. Sixty Chinese heterosexual women were recruited via Credamo (*M*_age_ = 30.30 years, *SD* = 8.94) and presented with five RIB measures (two scenarios and three pictures) in random order. After completing each measure, they were asked to answer three questions, which are “How much do you feel you have merged yourself into the scene just now?,” “How much do you believe the situation depicted will actually happen to you or to other women?,” and “How much do you feel that you have experienced a similar situation before or will experience one in the future?” (1 = *not at all*, 7 = *very much*). For each RIB measure, participants selected scores greater than 4 (i.e., the median score of the scale) for all three questions (*p*s < .001), indicating that they were almost able to relate these hypothetical scenarios (depicted either in text or cartoon figures) to their everyday experiences. These results confirm the ecological validity of our self-designed RIB measures to some extent.

4 We selected cartoon pictures to prevent the physical attractiveness of the man from influencing the results.

5 Before this study, a pilot study including 60 Chinese heterosexual women (*M*_age_ = 31.97 years, *SD* = 8.14) was conducted to check the effectiveness of this manipulation method. Participants’ state levels of self-objectification were measured with SOQ and SOBBS (α = .96) after the same writing task. The results revealed that those in the self-objectification group (*N* = 30) scored significantly higher on both SOQ and SOBBS compared to those in the control group (*N* = 30): *M* = 1.87, *SD* = 14.68 vs. *M* = –6.47, *SD* = 12.46; *t*(58) = 2.37, *p* = .021, Cohen’s *d* = 0.61 (SOQ); *M* = 3.38, *SD* = 0.76 vs. *M* = 2.60, *SD* = 0.82; *t*(58) = 3.83, *p* < .001, Cohen’s *d* = 0.99 (SOBBS), substantiating the effectiveness of this manipulation in inducing state self-objectification.
