# Supplementary material for: Love Through a Distorted Lens: The Role of Self-Objectification in Interpreting Ambiguous Female–Male Interactions as Romantic among Women
Source: Arch Sex Behav. 2026 Jan 13;55(1):409–25. doi: 10.1007/s10508-025-03358-1 (PMC12916924; doi:10.1007/s10508-025-03358-1)
Supplement: Supplementary file 1 — Supplementary file1 (DOCX 20 KB) [file 10508_2025_3358_MOESM1_ESM.docx]

**Supplementary Materials: The Assessment of the Potential Multicollinearity in Study 1**

We used the variance inflation factor (VIF) to test the extent of multicollinearity among the variables used in the regression analyses. According to Table S1, the VIF of all variables in all models ranges from 1 to 2, which is lower than 2.5, indicating no severe multicollinearity (e.g., Adeboye et al., 2014).

**Table S1** Results of VIF of the variables included in regression models

| Variables | VIF in Model 1 | VIF in Model 2  (SOQ as the predictor) | VIF in Model 2  (SOBBS as the predictor) |
| --- | --- | --- | --- |
| relationship status | 1.37 | 1.38 | 1.38 |
| self-esteem | 1.87 | 1.94 | 1.95 |
| social desirability | 1.08 | 1.09 | 1.11 |
| self-perceived attractiveness | 1.86 | 1.99 | 1.98 |
| BMI | 1.06 | 1.07 | 1.06 |
| SOQ / SOBBS |  | 1.10 | 1.11 |

**Reference**

Adeboye, N. O., Fagoyinbo, I. S., & Olatayo, T. O. (2014). Estimation of the effect of multicollinearity on the standard error for regression coefficients. *Journal of Mathematics*, *10*(4), 16–20. https://doi.org/10.9790/5728-10411620
